# Evaluation of Regulatory B Cell Subpopulations CD24++CD38++, CD24++CD27+, Plasmablasts and Their Correlation with T Regs CD3+CD4+CD25+FOXP3+ in Dialysis Patients and Early Post-Transplant Rejection-Free Kidney Recipients

**DOI:** 10.3390/jcm13113080

**Published:** 2024-05-24

**Authors:** Ariadni Fouza, Asimina Fylaktou, Anneta Tagkouta, Maria Daoudaki, Lampros Vagiotas, Efstratios Kasimatis, Maria Stangou, Aliki Xochelli, Vasiliki Nikolaidou, Georgios Katsanos, Georgios Tsoulfas, Lemonia Skoura, Aikaterini Papagianni, Nikolaos Antoniadis

**Affiliations:** 1 Department of Transplant Surgery, Center for Research and Innovation in Solid Organ Transplantation School of Medicine, Aristotle University of Thessaloniki, Ippokratio General Hospital, 54642 Thessaloniki, Greece; lampisv@yahoo.gr (L.V.); katsanosg@auth.gr (G.K.); tsoulfas@auth.gr (G.T.); nikanton@auth.gr (N.A.); 2 National Peripheral Histocompatibility Center, Department of Immunology, Ippokration General Hospital, 54642 Thessaloniki, Greece; fylaktoumina@gmail.com (A.F.); aliki.xochelli@gmail.com (A.X.); basoniko@hotmail.com (V.N.); 3 Laboratory of Biological Chemistry, School of Medicine, Aristotle University of Thessaloniki, 54124 Thessaloniki, Greece; taganneta@hotmail.com; 4 Department of Hygiene, Social-Preventive Medicine & Medical Statistics, School of Medicine, Aristotle University of Thessaloniki, 54124 Thessaloniki, Greece; 5 1st Department of Nephrology, School of Medicine, Aristotle University of Thessaloniki, Ippokration General Hospital, 54642 Thessaloniki, Greece; frasci@outlook.com.gr (E.K.); mstangou@auth.gr (M.S.); aapapagi@auth.gr (A.P.); 6 Microbiology Laboratory, Department of Immunology, AHEPA University Hospital, 54636 Thessaloniki, Greece; lemskour@auth.gr

**Keywords:** kidney transplantation, CD24++CD38++ (tBregs), CD24++CD27+ (mBregs), plasmablasts, CD3+CD4+CD25+FoxP3+ (Tregs)

## Abstract

**Background:** B and T regulatory cells, also known as Bregs and Tregs, are involved in kidney transplantation. The purpose of this study is to monitor changes in the frequency and absolute numbers of Tregs (CD3+CD4+CD25+FoxP3+), transitional Bregs (tBregs) (CD24++CD38++), memory Bregs (mBregs) (CD24++CD27+), and plasmablasts before (T0) and six months (T6) after transplantation. Additionally, we aim to investigate any correlation between Tregs and tBregs, mBregs, or plasmablasts and their relationship with graft function. **Methods:** Flow cytometry was used to immunophenotype cells from 50 kidney recipients who did not experience rejection. Renal function was assessed using the estimated glomerular filtration rate (eGFR). **Results:** At T6, there was a significant decrease in the frequency of Tregs, plasmablasts, and tBregs, as well as in the absolute number of tBregs. The frequency of mBregs, however, remained unchanged. Graft function was found to have a positive correlation with the frequency of tBregs and plasmablasts. A significant correlation was observed between the frequency and absolute number of tBregs only when the eGFR was greater than 60 but not at lower values. At an eGFR greater than 60, there was a positive correlation between the absolute numbers of Tregs and mBregs but not between Tregs and tBregs. No correlation was observed for any cell population in dialysis patients. **Conclusions:** The data show a correlation between the frequency and absolute number of tBregs and the absolute number of Tregs and mBregs with good renal function in the early post-transplant period.

## 1. Introduction

A thorough understanding of the different subpopulations of B cells in peripheral blood serves as an indicator of an individual’s immune status and provides insight into the contribution of B cells in transplantation. B cells play a crucial role in humoral responses, antigen presentation, and immune modulation and are therefore actively involved in renal graft rejection and tolerance.

After maturation in the periphery, B cells can be classified into four types: transitional, naive, memory cells, and plasma cells [1]. Transitional cells are recent emigrants from the bone marrow and have no antigen experience. They develop into mature B cells [2]. Naive B cells are mature B cells that have not encountered their cognate antigen. Upon activation, they differentiate into memory B cells, plasmablasts (precursors of plasma cells), and plasma cells [3].

Upon antigen challenge, human memory B cells undergo reactivation, proliferation, and differentiation into antibody-secreting plasmablasts. These plasmablasts are characterized by the CD27++CD38++ IgD+/− phenotype [3,4] and eventually develop into mature plasma cells [5]. Plasmablasts and memory B cells are crucial in transplantation, as they produce donor-specific antibodies (DSAs) for several years after [2]. Memory B cells can be classified into non-class switched memory cells (IgD+CD27+), class switched memory cells indicating previous antigen exposure (IgD-CD27+), and double-negative cells (IgD-CD27-) based on the expression of IgD and the key memory marker CD27 [3]. Naive cells are recognized as IgD+CD27− [6]. A diverse population of immunosuppressive cells, commonly referred to as B cells with regulatory properties (Bregs), has been identified at various stages of B cell maturation. There is no consensus on the definition and classification of these cells [7]. It is not possible to categorize them as a distinct population, because they lack a specific surface marker or transcription factor and are phenotypically heterogeneous, so multiple phenotypes have been studied [7]. Bregs stimulate the proliferation of Tregs; enhance their activity; and suppress the Th1, Th2, and Th17 subpopulations [8,9,10]. The production and secretion of the anti-inflammatory cytokine IL-10 is the most well-defined and studied property of Bregs [11,12,13,14]. There is mounting evidence that Bregs and Tregs, which exhibit a CD3+CD4+CD25+FoxP3+ phenotype [15], are interconnected and have a positive impact on transplant outcomes. As both categories facilitate immune tolerance and promote prolonged survival and allograft acceptance [10,16], two subsets of human regulatory B cells have attracted most of the research attention due to their distinct and shared characteristics [17]. These subsets are the immature transitional Bregs, tBregs, CD19+CD24++CD38++, first identified by Blair and colleagues in 2010 [18], and the memory Bregs, mBregs, CD19+CD24hiCD27+, equivalent to murine B10 cells, first characterized by Tedder’s group [11], which are the major B cells producing IL-10. The phenotypic characterization of the tBregs and mBregs populations through the utilization of surface markers does not provide an accurate indication of the number of cells exhibiting true immunoregulatory properties. In order to ascertain the number of cells with such characteristics within the populations under investigation, their ex vivo stimulation is employed, resulting in the production of cytokines such as IL-10 and probably IL-35 and transforming growth factor-β.

While there is a significant amount of literature on T cells and solid organ transplantation, research on Bregs and transplantation is not as well established. The variability of the published results makes it difficult to gain a clear understanding of the issue. Furthermore, the distribution of circulating Bregs and plasmablasts in rejection-free kidney transplant recipients remains unclear.

Therefore, we conducted a prospective cohort study on rejection-free kidney transplant recipients to investigate whether there is a difference in the frequency and number of peripheral B cells with regulatory properties, including plasmablasts, and Tregs six months after transplantation compared to before. Additionally, we evaluated the association of tBregs, mBregs, or plasmablasts with Tregs and their association with graft function.

## 2. Materials and Methods

### 2.1. Study Patients

The study cohort included fifty (*n* = 50) kidney transplant recipients who were free of rejection (with normal creatinine levels and a glomerular filtration rate and no clinical evidence of renal dysfunction) participated in the study.

Patients who underwent kidney transplantation were assessed at the time of transplantation, T0, and then again at 6 months post-transplantation, T6.

#### 2.1.1. Inclusion Criteria

Eligible participants were adults between the ages of 18 and 60 years who had been regular attendees at a nephrology outpatient clinic for at least two years prior to transplantation.

#### 2.1.2. Exclusion Criteria

Patients were excluded from the study if they had a history of malignancy, autoimmune disease, hematological disease, or treatment with monoclonal antibodies against B or T lymphocytes for less than 5 years. Those who had a cytomegalovirus or bacterial infection within the previous 3 months were also excluded, as were those who had an acute deterioration in renal function of unknown cause and/or who were followed for less than 2 years. Recipients from deceased donors with cardiac arrest were also excluded, as were patients who did not comply with treatment instructions.

### 2.2. Study Schedule

All recipients of a kidney transplant were considered to be eligible for the study on the basis of the above-mentioned inclusion criteria. Patients were included if they were on dialysis or preemptive (5%). The day of transplantation was considered the day of enrolment and defined as T0. Demographic and clinical information, as well as details on medical history, primary disease, and treatment, were extracted from the patients’ medical records (Table 1).

Blood samples were taken for laboratory and immunological evaluation at T0, before transplantation and before any immunosuppressive therapy, and at 6 months after transplantation, T6. Renal function medications were recorded. Renal function was evaluated using the eGFR using CKD-EPI (2021 update, (Chronic Kidney Disease Epidemiology Collaboration) formula.

Fifty-seven (57) patients were initially enrolled, of which seven patients withdrew from the study during the follow-up period, leaving a total of fifty patients in the study. Three patients had missing data in their medical records, two patients had a relapse of the primary disease, and two patients had an infection during the follow-up period.

### 2.3. Ethics Approval and Consent to Participate

The study was conducted in accordance with the Decleration of Helsinki and approved by the Ethics Committe of the Medical School of the Aristotle University of Thessaloniki (protocol code 4356, date of approval 26 January 2021). 

Written informed consent was obtained from each patient.

### 2.4. Immunosuppresion Regimen

The maintenance immunosuppressive regimen was the same for all patients according to the protocol of the transplant center, consisting of a combination of steroids, calcineurin inhibitor (tacrolimus), and antiproliferative agent (mycophenolate mofetil). Induction therapy consisted of an anti-CD25 antibody (basiliximab) or antithymocyte globulin. Basiliximab was used in 85% of patients.

### 2.5. Flow Cytometry of B Cell Subpopulations

Flow cytometric analysis of the circulating populations of tBregs, mBregs, plasmablasts within the total B cells, and the Treg population within the total CD3+CD4+ was performed to assess whether transplantation affected the populations studied by comparing their frequencies and absolute numbers at T0 and T6 in rejection-free kidney transplant recipients.

Blood samples were collected at T0 and T6 and were immediately tested. They were stained with the following monoclonal antibodies conjugated to fluorochromes for identifying B cells and their subpopulations:

anti-CD19 PC5.5, clone: J3-119, Beckman Coulter Inc, Sykesville, MD, USA;

anti-CD27 PE-Dylight 594, clone: LT27, EXBIO, Praha SA, Czech Republic;

anti-CD45-PC7, clone: J33, Beckman Coulter Inc, Sykesville, MD, USA;

anti-CD38-PB, clone: LS198-4-3, Beckman Coulter Inc, Sykesville, MD, USA;

anti-CD24-APC-Cy7, clone: SN3, EXBIO, Praha SA; Czech Republic No.: 25548611;

anti-IgD, clone: IA6-2, Fisher Scientific, Thermo Scientific LSG—Lagoas Park, Porto Salvo, Portugal

Flow cytometric analysis was performed immediately after sample preparation using a Navios EX flow cytometer (Beckman Coulter Inc, Sykesville, MD, USA)

**Figure 1:**
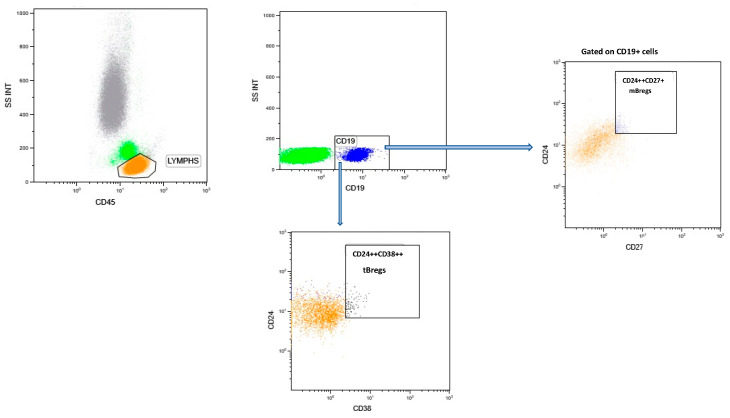
Gating strategy for B cell subpopulations. Representative flow cytometric data. CD45 and side scatter gating for lymphocytes. Total B cells are CD19+ cells, which are then analyzed for tBregs and mBregs cells.

Before cytometric acquisition and cell analysis, compensation was assessed using beads. Duplicate cells were excluded by plotting the forward scatter height versus forward scatter area, and individual cells were classified as monocytes, lymphocytes, and granulocytes based on forward and side scatter characteristics. All experiments were analyzed by forward scatter/side scatter gating on lymphocytes, excluding dead or dying cells. Gating was performed manually by a single operator. Figure 1 illustrates the gating strategy employed for B cell subpopulations.

The complete circulating population of B cells was recognized as CD19+. Moreover, transitional regulatory B cells (tBregs) were identified as CD19+CD38++CD24++ and memory regulatory B cells (mBregs) as CD19+CD24++CD27+. Plasmablasts have been identified as CD19+CD38++ CD27++ IgD+/− cells and total memory B cells as CD19+CD27+IgD+/−. Frequencies were determined relative to CD19+.

#### Flow Cytometry for Treg Identification and Intra-Cellular Staining of FOXP3

The following fluorochrome-conjugated monoclonal antibodies were used for the identification of Treg CD4+CD25+FOXP3+:

CD45-PC7, clone: J33, Beckman Coulter Inc., Sykesville, MD, USA

CD4-FITC, clone: 13B8.2, Beckman Coulter Inc., Sykesville, MD, USA 

CD25-PC5, clone: B1.49.9, Beckman Coulter Inc., Sykesville, MD, USA 

FOXP3-PE, clone: 259D, Beckman Coulter Inc., Sykesville, MD, USA

For intracellular staining of forkhead box protein 3 (FoxP3), peripheral blood mononuclear cells (PBMCs) were isolated from peripheral blood by Ficoll density gradient centrifugation. Erythrocyte lysis was performed, and PBMCs were washed with PBS. After surface staining, the cells were fixed and permeabilized for intracellular staining. PBMCs were then stained with fluorochrome-conjugated antibodies against intracellular FOXP3. Frequencies were determined relative to CD4 and to CD3+ T cells.

Patients’ absolute cell counts were calculated by combining flow cytometric proportions and lymphocyte counts using an automated hematology analyzer.

### 2.6. Statistics

For quantitative variables, normally distributed data were presented as the mean [standard error of the mean (SEM)], non-normal data as the median [interquartile range (IQR)], and categorical data as absolute values and relative frequencies. Differences between two independent groups were calculated using Mann–Whitney *U* tests. A non-parametric Wilcoxon signed-rank test for paired samples was used to compare pre- and post-transplant measurements. Spearman’s rank-based correlation coefficient was used to assess correlations. *p*-values less than 0.05 were considered significant. Statistical comparisons were performed using the R programming language in R4 3.

## 3. Results

### 3.1. Circulating B Cells, tBregs, mBregs, Plasmablasts, and Tregs in Dialysis Patients (Just before Transplantation) and Changes in the Frequency and Absolute Numbers Induced in the above Populations Post-Transplant

A number of factors may influence the outcome of a transplantation. These include a sensitization to HLA antigens, detected by a panel of reactive antibodies (PRA), HLA mismatches, the type of donation, dialysis vintage, or the use of induction therapy.

At T0, the sample population consisted of dialysis patients who were candidates for kidney transplantation. These patients were grouped according to their PRA levels, with 39 patients having negative PRA (group 1) and the remaining 11 having positive PRA (group 2). A comparison of the frequencies and absolute numbers of tBregs, mBregs, Tregs, and plasmablasts between the two groups at T0 and T6 revealed no statistically significant differences. Upon division of the population study according to donation type, no statistical difference was observed between the tBregs, mBregs, Tregs, and plasmablasts of living donors, who are considered to be of low risk of immunogenicity (negative PRA, a low number of HLA mismatches, and a short duration of dialysis), and those of deceased donors, who are considered to be of high risk of immunogenicity (usually with positive PRA, a high number of HLA mismatches, and a long duration of dialysis). Only two of the candidates exhibited preformed DSA. To date, no de novo DSAs have been identified. Furthermore, no statistically significant differences were observed in the number of HLA mismatches between the two groups.

At T6, kidney recipients showed a decrease in frequency and a statistically significant decrease in the absolute number of circulating total B cells, tBregs in both the frequency (2.3–0.2%, *p* < 0.01) and absolute number (1.5–0.1, *p* < 0.01), and frequency of plasmablasts (0.45–0.07, *p* < 0.01). It should be noted that plasmablasts are present at low levels in dialysis patients (Table 2). No significant changes were observed in the frequency (1.9–2.0%, *p* = 0.69) and absolute number (2.0–2.1, *p* = 0.63) of mBregs. However, a decrease in the frequency (5.2–4.7%, *p* = 0.05) of Tregs was observed, along with a significant increase in their absolute number (26–32, *p* < 0.01), as presented in Table 2.

### 3.2. Kidney Function in Relation to Frequency and Absolute Numbers of tBregs, mBregs, Plasmablasts, and Tregs in Kidney Transplant Recipients

We looked at the correlation between the frequencies and absolute numbers of the tBregs, mBregs, plasmablasts, and Tregs and the median GFR value (Table 3).

At T6, the tBregs showed a significant positive correlation with the eGFR in both frequency and absolute numbers (r = 0.47, *p* < 0.001 for frequency and r = 0.5, *p* < 0.001 for absolute numbers), whereas the plasmablasts only correlated with the eGFR in frequency (r = 0.36, *p* = 0.008), as shown in Table 3. In contrast to tBregs, there was no correlation between mBregs and the eGFR when analyzing frequencies (r = 0.17, *p* = 0.2379) and absolute numbers (r = 0.11, *p* = 0.28). There was also no correlation between Tregs and the eGFR, as shown by a Pearson’s correlation coefficient (normal distribution) of r = 0.041, with a *p*-value of 0.79 for frequency and r = 0.035 with a *p*-value of 0.87 for the absolute numbers.

Therefore, tBregs and plasmablasts are associated with renal function, whereas mBregs and Tregs are not.

### 3.3. Association of the Frequency and Absolute Number of tBregs with Different eGFR Values (Grading of Graft Function)

Based on the statistically significant association between tBregs and eGFR, tBregs from kidney transplant recipients (*n* = 50) were divided into two groups, group 1 and group 2, according to their eGFR. Group 1 (*n* = 20) consisted of individuals with an eGFR > 60 mL/min/1.73 m^2^, whereas group 2 (*n* = 30) consisted of individuals with an eGFR ≤ 60. Both the frequency and absolute number of tBregs were higher in group 1 compared to group 2, with *p*-values of 0.0008 and 0.002, respectively. There was no statistically significant correlation found between Tregs and mBregs when grouping patients based on their eGFR. The data suggest that a higher frequency and absolute number of tBregs, although numerically low, are associated with higher eGFR values. This indicates that tBregs may play a role in maintaining good renal function six months after transplantation.

### 3.4. Association of the Circulating B Cell Subpopulations with Tregs in Dialysis Patients and after Their Transplantation

In the correlation analysis of tBregs, mBregs, and plasmablasts with Tregs, the sample size was limited to 43 patients, because the Tregs data were only available for this number of patients. There was no correlation observed between the frequency (r = −0.10, *p* = 0.5) or absolute number (r = 0.081, *p* = 0.6) of Tregs and tBregs in dialysis patients. After transplantation, a similar correlation trend was observed between Tregs and tBregs in terms of frequency (r = −0.007, *p* = 0.96) and absolute numbers (r = 0.003, *p* = 0.98); see Table 4. There was no correlation found between the frequency and absolute numbers of Tregs and mBregs at both T0 and T6, as shown in Table 4.

**Figure 2:**
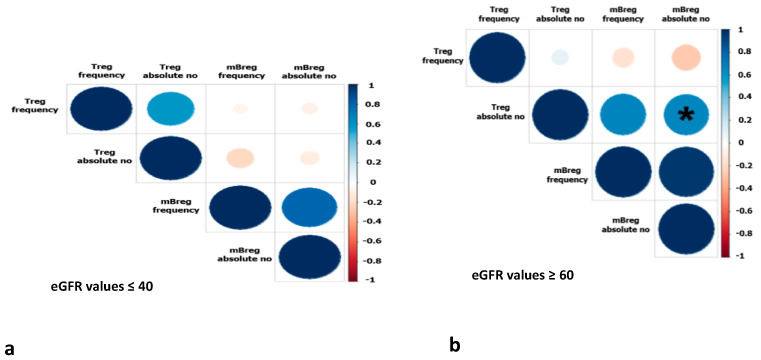
Correlation matrix between the absolute numbers (no) and frequency of mBregs and Tregs of transplanted patients at T6 with (**a**) eGFR ≤ 40 and (**b**) eGFR ≥ 60. A significant correlation is shown with an asterisk.

Furthermore, we investigated whether there was an association between Tregs and tBregs or mBregs at different eGFR values after transplantation (values ≥ 60 mL/min/1.73 m^2^ indicate good graft function and ≤40 mL/min/1.73 m^2^ indicate fair to poor graft function). A significant correlation (r = 0.65, *p* = 0.002) was found between the absolute numbers of Tregs and mBregs in recipients with an eGFR equal to or greater than 60 (Figure 2). However, there was no significant correlation for frequency (r = −0.15, *p* = 0.522). When the eGFR was less than 40, there was no significant association between the eGFR and either frequency (*p* = 0.08) or absolute numbers (*p* = 0.53).

There was no correlation found between Tregs and tBregs in terms of frequency (r = −0.05, *p* = 0.788) or absolute numbers (r = −0.1, *p* = 0.629) when the eGFR was equal to or greater than 60. Similar results were obtained at GFR values ≤ 40. Furthermore, when plasmablasts were correlated with Tregs at different eGFR values, similar results were obtained.

Kidney transplant recipients were also categorized according to the type of donor, either living or deceased, to show whether the type of graft donation would affect the immunophenotypes of different regulatory B cell subpopulations and Tregs. We did not find any association between the frequencies and the absolute numbers of any of the B cell subpopulations tested, nor for the Tregs. The subsets of B cells that differed significantly between time points or groups did not correlate with the age or gender of the recipients, as well as the dialysis vintage.

In this cohort study, we investigated the effect of induction therapy on different B regulatory subpopulations and Tregs. The results suggest that there was no statistically significant difference in the frequency or absolute numbers of the cell populations tested.

## 4. Discussion

Knowledge of the distribution of B cell subsets in the blood may reflect an individual’s immune status and may help to understand B cell involvement in transplantation. Bregs are important in kidney transplant recipients due to their suppressive function, potential tolerance-inducing power through both IL-10-dependent and IL-10-independent mechanisms, and potential protective role in systemic renal diseases such as graft acceptance and reducing DSA production [12]. Breg cells are known to suppress T cells and are able to induce Tregs through the conversion of CD4+CD25− cells [19]. However, the relationship between Bregs and Tregs in transplantation is not well understood.

We conducted a study to investigate the relationship between Bregs and Tregs in transplant candidates who were on dialysis and 6 months after their kidney transplant. The kidney recipients are all on the same immunosuppressive therapy, which includes steroids, tacrolimus, and MMF. They have not experienced any rejection. It cannot be ruled out that a subclinical rejection may have occurred. This could be substantiated by the implementation of protocol biopsies, which are not applicable in our center.

The study evaluated the changes in the frequency and absolute numbers of tBregs, mBregs, plasmablasts, and Tregs after transplantation. Correlations between the different populations were examined, as well as their association with graft function.

Consistent with the findings of Wang L et al. [20], we observed a slight decrease in the frequency and a significant decrease in the absolute number of total circulating B cells at T6. The frequency and absolute number of tBregs showed a statistically significant decrease, consistent with the findings of Schuller et al. [21], although their patients were sampled one year post-transplant. As transplanted patients continue to receive high doses of immunosuppressants six months after transplantation, the observed decrease in tBregs may be due to corticosteroid-induced apoptotic effects on tBregs. These cells are immature and more sensitive to corticosteroids [22]. In addition, dialysis patients often have peripheral B cell abnormalities, which may contribute to these findings [20]. Chung et al. [23] found that tacrolimus and mycophenolate mofetil reduced the frequency and number of immature B cells in in vitro cultures, which may explain our findings of a reduction in tBregs due to general immunosuppressive therapy. Ibrahim et al. [24] observed quantitative changes in tBregs. They studied the cell population over a longer period and found that tBregs decreased in less than a year in 45 patients in their cohort. An observed decrease in plasmablasts was confirmed by Schuller M et al. [21]. There was no change in the mBregs, possibly due to the high frequency of memory B cells in the peripheral blood (see Table 2).

In kidney recipients, immunosuppressive therapy affects B and T cells differently. Our findings show that Tregs are outnumbered compared to Bregs. The decrease in tBregs may be compensated for by an increase in Tregs at T6. A significant increase in the absolute number of Tregs, possibly due to IL-10+Bregs, was observed in the total population of tBregs and mBregs in our cohort. Although we did not determine the IL-10+Bregs population, we believe that these cells are likely redirecting T cell differentiation in favor of Tregs and promoting their proliferation. A larger sample size and a functional study would be required to confirm this finding and to investigate the expression of IL-10 within tBregs and mBregs. However, ex vivo functional assays for Bregs are challenging, making it difficult to determine the most equivalent conditions to in vivo conditions.

Induction therapies did not affect our results regarding Bregs and Tregs.

It was anticipated that significant differences in the peripheral Tregs would be observed when the two groups were compared, given the mechanism of action of ATG. This is due to the depletion of CD4 T cells in the ATG group. Given that ATG does not affect thymic function, it is likely that there is a reconstitution of T cells at six months post-transplantation. Consequently, at this assessment time, no significant changes were observed. With regards to Bregs, given the crosstalk between immune cells and probably between them and Tregs, no changes were also observed.

In contrast to the findings of Lattore et al. [25], we observed a numerical decrease in Tregs with CNI treatment compared to the pre-transplant levels. This may be due to the fact that they compared their results to those of healthy individuals rather than pre-transplant values, and the study period was different. Regarding the absolute number of Tregs, our results were not in agreement with those of Aly MG et al. [26], who reported a lower absolute number of Tregs compared to the pre-transplant levels.

A significant correlation was found between the frequency and absolute number of tBregs and eGFR (Spearman’s correlation coefficient, r = 0.47, *p* < 0.001 for frequency and r = 0.5, *p* < 0.001 for absolute number). Our results are in contrast to those of Shabir et al. [27], who found no association between tBregs frequency and eGFR 12 months after transplantation. However, Imrahim EH et al. [24] found that only the frequency of tBregs was associated with the eGFR, although not significantly, but not the absolute number. We found a significant correlation between the eGFR and the frequency of plasmablasts, suggesting that only the frequency of plasmablasts and both the frequency and absolute number of tBregs are associated with renal function. A significant correlation was found between graft function and tBregs, both in terms of frequency and absolute number, at an eGFR greater than 60 but not at lower eGFR values. The higher frequency and absolute number of tBregs at this eGFR level may suggest that tBregs could potentially contribute to good renal function six months after transplantation by secreting cytokines that improve or maintain renal function. It has been postulated that tBregs may contribute to the maintenance of renal function through the production of IL-10. This immunoregulatory cytokine induces regulatory T cells (Tregs), which play a role in the development of tolerance and the preservation of graft function. Furthermore, Tregs secrete IL-10, which may influence tBregs and mBregs to sustain IL-10 production, thereby creating an anti-inflammatory environment that facilitates the maintenance of good graft function. This hypothesis is further supported by the findings of a significant correlation between the absolute numbers of Tregs and mBregs at eGFR values >60. It is also possible that TGF-β may contribute to the anti-inflammatory environment. Additionally, within the tBregs population, there are B regulatory cells that express IL-10 and B effector cells that express TNF-α with proinflammatory properties. For the anti-inflammatory environment to persist and maintained good graft function, the number of B regulatory cells must exceed that of B effector cells [28].

The study investigated the potential interaction between tBregs, mBregs, and Tregs in the immune system, focusing on their frequencies and absolute numbers. No correlation was found between the frequencies and absolute numbers of tBregs and Tregs in dialysis patients, either before or after transplantation. Ibrahim et al. [24] and Piloni et al. [29] reported similar results. However, it is important to note that Piloni’s study focused on lung transplantation and not on kidney transplantation.

The available information on mBregs in transplant patients is limited [28]. Zhou et al. [30] found that reduced numbers of circulating mBregs may be associated with liver graft rejection. Although there is a paucity of data concerning B regulatory cells in other transplanted organs, there is strong evidence that tBregs and mBregs cells play a role in promoting tolerance in liver and lung transplantation.

In our study group, the frequency and absolute number of mBregs remained unchanged compared to the pre-transplant levels. However, we observed a significant correlation between the absolute number of mBregs and Tregs only in cases of good graft function, as indicated by an eGFR of >60 mL/min/1.73 m^2^, but not in cases of lower eGFR values of <40 mL/min/1.73 m^2^. To our knowledge, this study is the first to demonstrate a correlation between mBregs and Tregs populations that may contribute to optimal graft function.

The limitations of our study include the use of a single-center sample population, the observational nature of the study in a homogeneous population with a modest sample size, the relatively short follow-up for the measurement of immune cell subsets after transplantation, and the focus of the analysis on peripheral blood. This issue could be addressed by participation in multicenter trials, which would also allow for sample diversity and the consideration of different immunosuppressive protocols. Another potential avenue for investigation could be renal biopsies, with the profile of lymphocytes detected being compared with those from peripheral blood. The functional assessment of the cell populations studied was not included but will be investigated in the future.

A longitudinal study is underway at the following one- and two-year time points to assess any changes in the cell subsets studied and to determine how these changes affect longer-term renal function. These changes will be further evaluated in relation to the donation type, duration of hemodialysis, recipient sensitization status, immunosuppressive therapy (induction and maintenance), and any de novo DSA formation.

A comparison of the results obtained at 6 months post-transplantation with cellular population studies of long-term recipients (10–20 years post-transplantation) will help to determine which populations are crucial for tolerance and protection against chronic rejection, thus ensuring long-term graft survival.

In conclusion, this study shows that tBregs and plasmablasts decrease early after transplantation and are associated with good graft function independently of Tregs. In addition, there is statistical evidence of a positive correlation between the absolute number of mBregs and Tregs in good renal graft function (eGFR of >60 mL/min/1.73 m^2^) six months after transplantation. Notably, this correlation between memory B cells and Tregs was not observed in pre-transplant dialysis patients.

## Figures and Tables

**Table 1:** Study group demographics and clinical characteristics. HD: Hemodialysis. CAPD: continuous ambulatory peritoneal dialysis.

Study Sample N:50	
sex	Female:16 Male:34
Age in years	51 (28.2, 49.6)
Type of donor	Deceased, brain death: 36 (72%) Living: 14 (28%)
Duration of dialysis (months)	89 (13.2, 65.1)
Type of dialysis	HD: 40 (85%) CAPD: 6(%) CAPD + HD: 4(9%)
Delayed graft function	Yes: 12 (24%) No: 38 (76%)
Cold ischemia time	19.2 (4.6, 14.2)
Distribution of underlying kidney disease	
Polycystic kidney disease%	21%
Primary glomerulopathies%	22%
Reflux nephropathy%	13%
Diabetes mellitus%	4%
Nephrosclerosis/hypertension%	4%
Urinary tract infections/ stones%	3%
Other%	18%
Unknown%	15%
Induction therapy:	
Basiliximab%	82% (41 patients)
ATG%	18% (9 patients)
Maintanance immune suppression:	
tacrolimus/mycophenolate/prednisone%	100.0%
eGFR (mL/min/1.73 m^2^) six months after transplantation	52 (36–89)

**Table 2:** Differences in phenotypes of B cell subsets and Tregs in kidney recipients at T0 and T6. Values that were significantly different in the two groups were indicated by their *p*-value. Mediana are in (IQR) percentages (% cells), and absolute numbers of cells are expressed as cells/μL.

Cell Populations	Τ0	Τ6	*p* Significance *p* < 0.05
	*Ν* = 50 Median (IQR)	*Ν* = 50 Median (IQR)	
CD19+, total B cells percentage (% cells) CD19+, total B cells absolute number	8 (5.9–12.0) 0.08 (0.01–0.39)	7.4 (5.0–9.1) 0.01 (0.001–0.040)	0.02 <0.01
tBregs percentage (% cells) tBregs absolute number	2.3 (0.3–5.8) 1.5 (0–5)	0.2 (0–1) 0,1 (0–1)	<0.01 <0.01
mBregs percentage (% cells) mBregs absolute number	1.9 (0.3–5/0) 2 (0–5)	2 (0.3–4.4) 2 (0–3.7)	0.69 0.63
plasmablasts percentage (% cells) plasmablasts absolute number	0.45 (0–1.7) 0 (0–0)	0.07 (0–0.8) 0 (0–0)	<0.01 0.33
Tregs percentage (% cells) Tregs absolute number	5.2 (4.2–6.6) 26 (15.5–32.5)	4.7 (3.9–5.3) 32 (24.5–43.5)	0.05 <0.01
Total memory percentage (% cells) Total memory absolute number	24.7 (15.4–34.5) 0.39 (0.03–1.10)	28 (21.8–42.0) 0.08 (0.003–0.300)	0.28 <0.01

**Table 3:** Correlation coefficients of the association of tBregs (frequency and absolute numbers), mBregs (frequency and absolute numbers), plasmablasts (frequency and absolute numbers), and Tregs (frequency and absolute numbers) with the median eGFR value. Values of *r* were calculated using Spearman’s rank correlation (*) and Pearson’s rank correlation (**) tests; nd: not determined.

Cell Populations	eGFR	
	r	*p*
Frequency of tBregs %	0.47 *	<0.001
Abs no of tBregs	0.50 *	<0.001
Frequency of mBregs%	0.17 *	0.237
Abs no of mBregs	0.18 *	0.223
Frequency of plasmablasts %	0.36 *	0.008
Abs no of plasmablasts	nd	nd
Frequency of Tregs %	0.041 **	0.79
Abs no of Tregs	0.044 **	0.75

**Table 4:** A non-significant association of the frequencies and absolute numbers of tBregs and mBregs with the frequencies and absolute numbers of Tregs was found in transplanted patients at T6, as shown by the correlation coefficient. %: % frequency, Abs: absolute number.

T0	T0	T0	T6	T6	T0	T0	T6	T6
	%tBregs	Abs tBregs	% Bregs	Abs tBregs	%m Bregs	Abs mBregs	%m Bregs	Abs mBregs
	r	r	r	r	r	r	r	r
%Tregs	−0.10		−0.007		−0.1		−0.08	
Abs Tregs		0.081		0.003		0.03		0.21

## Data Availability

Upon request, the corresponding author can provide the data sets used and analyzed in this study.
